# Identification of the Anti*Listerial* Constituents in Partially Purified Column Chromatography Fractions of *Garcinia kola* Seeds and Their Interactions with Standard Antibiotics

**DOI:** 10.1155/2014/850347

**Published:** 2014-01-08

**Authors:** D. Penduka, L. Buwa, B. Mayekiso, A. K. Basson, A. I. Okoh

**Affiliations:** ^1^Applied and Environmental Microbiology Research Group (AEMREG), Department of Biochemistry and Microbiology, University of Fort Hare, P. Bag X1314, Alice 5700, South Africa; ^2^Department of Biochemistry and Microbiology, University of Zululand, P/Bag X1001, KwaDlangezwa 3886, KwaZulu-Natal Province, South Africa

## Abstract

Partially purified fractions of the n-hexane extract of *Garcinia kola* seeds were obtained through column chromatography and their constituents were identified through the use of gas chromatography coupled to mass spectrometry (GC-MS). Three fractions were obtained by elution with benzene as the mobile phase and silica gel 60 as the stationery phase and these were named Benz1, Benz2, and Benz3 in the order of their elution. The anti*Listerial* activities of these fractions were assessed through MIC determination and only Benz2 and Benz3 were found to be active with MIC's ranging from 0.625 to 2.5 mg/mL. The results of the GC-MS analysis showed Benz2 to have 9 compounds whilst Benz3 had 7 compounds, with the major compounds in both fractions being 9,19-Cyclolanost-24-en-3-ol, (3.**β**.) and 9,19-Cyclolanostan-3-ol,24-methylene-, (3.**β**.). The Benz2 fraction was found to have mainly indifferent interactions with ampicillin and penicillin G whilst mainly additive interactions were observed with ciprofloxacin. The Benz3 fraction's interactions were found to be 50% synergistic with penicillin G and 25% synergistic with ciprofloxacin and ampicillin. A commercially available 9,19-Cyclolanost-24-en-3-ol, (3.**β**.) was found not to exhibit any anti*Listerial* activities at maximum test concentrations of 5 mg/mL, suggesting that the compound could be acting in synergy with the other compounds in the eluted fractions of *Garcinia kola* seeds.

## 1. Introduction

Plants produce a vast diversity of secondary metabolites most of which are phytochemicals that have potential use in the pharmaceutical industry for new drug development purposes. Phytochemicals are naturally occurring bioactive plant compounds that act as a natural defence system for the host plant and also provide colour, aroma, and flavour [1]. Some phytochemicals have been shown to possess antimicrobial properties and these include terpenoids, essential oils, alkaloids, lectins, polypeptides, polyacetylenes, and phenolics, of which phenolics can be further divided into phenolic acids, flavonoids, quinones, tannins, coumarins, and simple phenols [2].

The terpenes are one of the largest and most diverse groups of plant secondary metabolites. They include complex compounds that are formed by the cyclization of 2,3-oxidosqualene [3]. They include terpenoids and sterols as well as essential oils which carry the fragrance of the plant. Terpenes possess antimicrobial properties and their mechanism of action is mainly through disruption of the bacterial membrane [2, 3].

Flavones, flavonoids, and flavonols are phenolic structures with one carbonyl group and are synthesized by plants in response to microbial infection [4, 5] and often have broad spectrum antibacterial activities *in vitro* [5, 6]. Flavonoids and flavones modes of action usually involve formation of complexes with cell walls, binding to adhesins and inactivation of bacterial enzymes [2]. Flavonoids are known to enhance the effects of vitamin C as well as having antioxidant properties. They are also biologically active against liver toxins, tumours, viruses, allergies, and inflammation [1].

Tannin is a general descriptive name for a group of polymeric phenolic substances capable of tanning leather or precipitating gelatin from solution, a property known as astringency, and they are found in almost every plant part [2, 7]. They exert their antimicrobial action in different ways which can include amongst others, binding to proteins, binding to adhesins, bacterial enzyme inhibition, substrate deprivation, formation of complexes with bacterial cell wall, membrane disruption, and metal ion complexation [2]. Alkaloids on the other hand intercalate into cell wall and/or DNA [2], causing leakage of bacterial cell contents or disruption of DNA synthesis, respectively, which eventually leads to bacterial death.

Continual research on bioactive substances from plants can be a possible lead to the discovery and formulation of new potent antibacterial compounds that could help alleviate the problem of antibiotic resistance [8]. *Garcinia* is a large genus of polygamous trees or shrubs, that can be found in tropical Asia, Africa, and Polynesia and is a rich source of bioactive molecules including xanthones, flavonoids, benzophenones, lactones, and phenolic acids amongst others [9].

*Garcinia kola* is a traditional medicinal plant that is cultivated and distributed throughout west and central Africa. Its medicinal uses include being an antiparasitic and antimicrobial agent as well as being a purgative. The seeds are traditionally used to prevent and relieve colic, cure head or chest colds, and relieve cough [10]. Several studies have shown the antibacterial potentials of different extracts of *Garcinia kola* seeds *in vitro* [11–13], whilst other studies have shown some therapeutic effects of the seeds in human clinical trials and in some animal models [14, 15]. A number of phytochemicals that can account for the antibacterial activities of *Garcinia kola* seeds have been identified in them and these include tannins, saponins, alkaloids, and cardiac glycosides. Biflavonoids such as kolaflavonone and 2 hydroxy flavonoids are the most abundant phytochemicals in the seeds [16].

The presence of 9,19-Cyclolanost-24-en-3-ol, (3.*β*.) also known as cycloartenol and 9,19-Cyclolanostan-3-ol,24-methylene-, (3.*β*.) also known as 24-methylenecycloartanol in *Garcinia kola* seeds extracts has been reported by other authors [17, 18], but, to the best of our knowledge, this is among one of the first papers that report the presence of these sterols amongst other compounds such as lanosterol and *β*-amyrin in the column chromatography eluted fractions of the n-hexane extract of *Garcinia kola* seeds that exhibited anti*Listeria *activities *in vitro*.

## 2. Materials and Method

### 2.1. *Listeria* Isolates and Plant Material

The *Listeria* isolates used in this study were previously isolated from wastewater effluents [19] and were kept in the bacterial culture collection of the Applied and Environmental Microbiology Research Group (AEMREG) University of Fort Hare Alice, South Africa. The *Garcinia kola* seed powder was also collected from the plant material collection of the above-mentioned research group.

### 2.2. Preparation of the Crude n-Hexane Extract

The extracts were prepared using the method of Basri and Fan [20] as outlined by Penduka and Okoh [21]. Briefly, the method involved the steeping of the seed powder in n-hexane solvent for 48 h with shaking in an orbital shaker at 50 rpm (Stuart Scientific Orbital Shaker, UK), followed by centrifugation at 3 000 rpm for 5 min (Beckman Model TJ-6RS Centrifuge, UK), filtration using a Whatman no. 1 filter paper, and subsequent evaporation of the solvent using a rotary evaporator (Steroglass S.R.L, Italy) at 50°C. The extract was thereafter dried to a constant weight in a fume cupboard.

### 2.3. Column Chromatography

The method of Selowa et al. [22], with some modifications, was used to elute the fractions during column chromatography. A suspension of silica gel and benzene was poured into a glass column (40 cm long × 2.5 cm diameter) up to a height of 30 cm being careful to prevent formation of gaps and bubbles and equilibrated with 100% benzene. An overnight dried mixture of seven grams of the n-hexane extract and 14 grams of the silica gel 60 (Merck, Germany; particle size 0.063 to 0.2 mm/70 to 230 mesh) in benzene solvent was loaded onto the column and eluted with 100% benzene first and then with the solvent combination of benzene : ethanol : ammonium hydroxide (B.E.A) (36 : 4 : 0.4 v/v) collected on the basis of solvent polarity and/or colour separation. The fractions were dried in a rotary evaporator after which they were dried to a constant weight in a fume cupboard. Thin layer chromatography (TLC) was carried out according to the descriptions of Eloff et al. [23] using the B.E.A solvent combination at a ratio 36 : 4 : 0.4 v/v such that fractions with similar profiles could be pooled together.

### 2.4. MIC Determination

The inoculum was standardised using the EUCAST [24] colony suspension method, whereby 24 h old cultures grown on nutrient agar plates were suspended in sterile saline solution (0.85% NaCl) to give a turbidity that is equivalent to a 0.5 McFarland standard, which was then diluted a hundredfold before use. The starting test concentrations of the fractions were prepared by dissolving them in acetone at 20% of the final volume which was then made up of sterile distilled water. The broth microdilution assay performed in 96-well microtiter plates was used to determine the MICs of the eluted fractions following the method of EUCAST [24] with some modifications. A starting concentration of 5 mg/mL of the test fraction was serially diluted two-fold in the microtitre plates to make 12 different test concentrations in double strength Mueller-Hinton broth to account for the dilution factor caused by the addition of the test fraction. A 20 *μ*L volume of the test *Listeria* strains was added into each well containing 100 *μ*L of the different test fractions' in broth. The 20% acetone was also included as a solvent control to ascertain if it had anti*Listeria* activities. Ciprofloxacin, ampicillin, and penicillin G dissolved in sterile distilled water were used as the positive controls. Sterility, growth, and absorbance control wells were also included, after which the plates were then incubated for 18–24 h at 37°C. An automatic ELISA microplate reader (SynergyMx BiotekR, USA) at 620 nm was used to measure the absorbance of the plates before and after incubation to determine the MIC. The MIC was defined as the lowest concentration of the test antibacterial agent that did not exhibit growth by calculating the difference in absorbance between the test wells and the control wells that had the broth and antimicrobial agent alone without the test bacteria.

### 2.5. Gas Chromatography Coupled to Mass Spectrometry (GC-MS)

An Agilent 6890N GC with CTC CombiPAL Autosampler and Agilent 5975B MS was used for the GC-MS analyses of the fractions using an Rtx-5MS (30 m, 0.25 mm ID, 0.5 *μ*m film thickness) Restek 12723-127 column. The test samples were dissolved in 1 mL dichloromethane and then derivatized with pyridine and MSTFA (*N*-Methyl-*N*-trimethylsilyltrifluoroacetamide) followed by a 30 min incubation period at 80°C before injection onto the GC column. The instrument settings were injector temperature 280°C, injection volume 1 *μ*L, split injection mode, split ratio of 15 : 1, carrier gas Helium, flow rate 1 mL/min, MS transfer 280°C, mode EI+; electron energy 70 eV, acquisition mode scan, scanning mass range from 40 to 550 *m/z*, and a solvent delay of 8 min. The initial oven temperature was at 150°C which was held for 1 min to a final temperature of 325°C which was then held for 20 min. The compounds were identified by comparison of their retention indices and mass spectra with those in the National Institute of Standards and Technology (NIST) library.

### 2.6. Determination of the Anti*Listerial* Activities of 9,19-Cyclolanost-24-en-3-ol, (3.Beta.)

The microtiter broth microdilution assay according to EUCAST [24] was used to determine the anti*Listerial* activities of the purchased standard 9,19-Cyclolanost-24-en-3-ol, (3.beta.) (ChromaDex). The descriptions of the method are as those explained above for the determination of the MICs of the eluted column chromatography fractions.

### 2.7. Interactions between Antibiotics and the Eluted Column Chromatography Fractions

The interactions of the column fractions and the selected test antibiotics were interpreted by using the fractional inhibitory concentration (FIC) indices which were determined using the chequerboard method according to the descriptions of Miranda-Novales et al. [25] with some modifications. The tests were performed in 96-well microtitre plates and each well contained 100 *μ*L of the test antimicrobial combination. Growth, sterility, and absorbance controls were also included in each plate. The inoculum suspension method of EUCAST [24] was used to prepare the test *Listeria* inoculums whilst the MICs were read after 18–24 h incubation at 37°C. An automatic ELISA microplate reader (SynergyMx BiotekR, USA) at 620 nm was used to measure the absorbance of the plates before and after incubation to determine the MIC. The FIC index of a column fraction (FIC_C_) was calculated as the ratio of the MIC value of the column fraction in combination over the MIC value of the column fraction alone, and the FIC index of the antibiotic (FIC_A_) was calculated as the ratio of the MIC value of the antibiotic in combination over the MIC value of the antibiotic alone. The overall FIC index (ΣFIC) was calculated as the summation of the FIC_C_ and the FIC_A_. The interactions were interpreted as synergism when the ΣFIC index ≤ 0.5, additive when ΣFIC index is between >0.5 and ≤1, and indifference when the ΣFIC index is >1 and ≤4 whilst antagonism was defined as when an ΣFIC index is >4 [26, 27].

### 2.8. Statistical Analysis

The Microsoft Office Excel 2007 version for Windows program was used to determine the means and standard deviations.

## 3. Results

### 3.1. MIC Determination

The MICs of the different eluted column chromatography fractions are as seen in Table 1. Three fractions were eluted by the benzene solvent named Benz1, Benz2, and Benz3; two out of the three fractions eluted by the benzene solvent exhibited anti*Listeria* activities. Benz2 fraction had MIC values ranging from 0.625–1.25 mg/mL depending on the* Listeria* species and Benz3 MIC values were ranging from 0.625–2.5 mg/mL depending on the *Listeria* species whilst Benz1 fraction did not exhibit any anti*Listeria* activities. The results of the column fractions eluted by the B.E.A solvent combinations are reported in Chapter Seven.

### 3.2. Thin Layer Chromatography

The thin layer chromatography showed one band on each of the three fractions. The band of Benz1 had an *R*_*f*_ value of 0.957 ± 0 and it was also visible at 365 nm UV wavelength. Benz2 band had an *R*_*f*_ value of 0.75 ± 0.009 and Benz3 had one band with an *R*_*f*_ value of 0.82 ± 0.004. Bands for both Benz2 and Benz3 were not visible under UV at both 365 nm and 302 nm.

### 3.3. GC-MS

**Figure 1:**
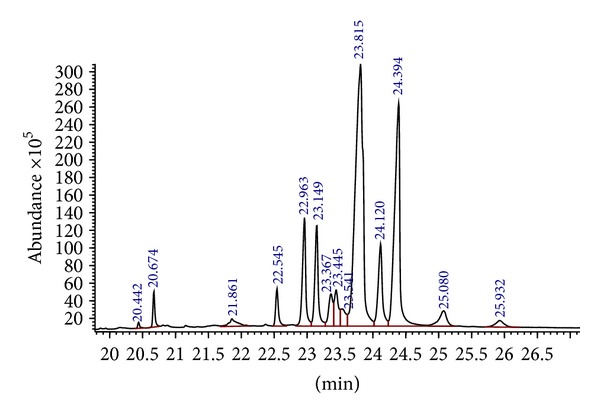
GC-MS chromatogram of the compounds in Benz2 column chromatography fraction of the n-hexane extract of *Garcinia kola* seeds.

**Figure 2:**
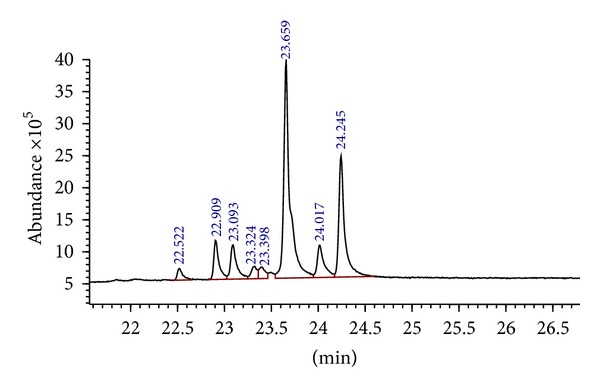
GC-MS chromatogram of the compounds in the Benz3 column chromatography fraction of the n-hexane extract of *Garcinia kola* seeds.

The GC-MS analysis was carried out for the two active fractions, namely, Benz2 and Benz3 and the GC-MS chromatograms are as shown in Figure 1 for Benz2 and in Figure 2 for the Benz3 fraction while Tables 2 and 3 show the compounds found in the Benz2 and Benz3 fractions, respectively. Combining all the identical compounds at different retention times Benz2 had a total of 9 compounds as 9,19-Cyclolanost-24-en-ol, (3.*β*.) was identified five times at different retention times and 4-methyl-2-trimethylsilyloxy-acetophenone was identified twice. Benz3 had a total of 7 compounds as 2,4,6-cycloheptatrien-1-one, 3,5-bis-trimethylsilyl was reported twice at different retention times. The compounds 9,19-Cyclolanost-24-en-3-ol, (3.*β*.) and 9,19-Cyclolanostan-3-ol,24-methylene-, (3.*β*.) were the most abundant in both fractions. 9,19-Cyclolanost-24-en-ol, (3.*β*.) had a total area percentage of 53.6% in Benz2 and of 46.0% in Benz3 after combining area of the compound at different retention times. The compound 9,19-Cyclolanost-3-ol, 24-methylene-, (3.*β*.) had a total area percentage of 23.4% in Benz2 and 24.7% in Benz3. The compound 4-methyl-2-trimethylsilyloxy-acetophenone was also common in both fractions.

### 3.4. Anti*Listerial* Activities of 9,19-Cyclolanost-24-en-3-ol, (3.*β*.)/Cycloartenol

The commercially obtained compound 9,19-Cyclolanost-24-en-3-ol, (3.*β*.) (ChromaDex) which was the most abundant compound in the fractions did not exhibit any anti*Listerial* activities against any of the test *Listeria* isolates at a maximum test concentration of 5 mg/mL.

### 3.5. Interactions of Benz2 Fraction with Some Test Antibiotics *In Vitro*

The interactions of the Benz2 fractions with the test antibiotics are as shown in Table 4. The interactions of the fraction and ciprofloxacin were 75% additive and 25% synergistic. The fraction's interaction with penicillin G was 25% additive and 75% indifferent, whilst all the interactions of the fraction and ampicillin were all indifferent.

### 3.6. Interactions of the Benz3 Fraction and the Test Antibiotics

Table 5 shows the results obtained for the interactions of the Benz3 fraction and the test antibiotics. Penicillin G had 50% synergistic and 50% additive interactions with the fraction Benz3. Ciprofloxacin had 25% synergistic and 75% additive interactions with the fraction, while the interactions of the fraction with ampicillin were 25% synergistic, 25% additive, and 50% indifferent.

## 4. Discussion

The activities portrayed by the Benz2 and Benz3 fractions against the test *Listeria* species in this study are of significance in anti*Listeria* chemotherapy because *L. monocytogenes* is pathogenic in humans and animals causing the disease listeriosis and *L. ivanovii* causes listeriosis in animals, whilst, on the other hand, there have been reported cases of human listeriosis being caused by *L. ivanovii* [28, 29] and *L. grayi* [30–32] species making them also potential human pathogens, especially in individuals with compromised immune systems.

Fractions Benz2 and Benz3 showed one band only on the TLC plate but, however, showed multiple compounds during GC-MS analysis. These results are similar to those observed by other authors such as Seanego and Ndip [33] who observed 10 compounds mainly fatty acids and their esters in a fraction of *Garcinia kola* seeds' methanol extract, that had shown only one band on the TLC plate. Floriani et al. [34] also identified three plant phytosterols in a fraction of *Epidendrum mosenii* stem's methanol extract that had shown only one band during TLC analysis. The observation of only one TLC band in the Benz2 fraction in our study could be attributed to the fact that the sterols identified in it, namely, 9,19-Cyclolanost-24-en-3-ol, (3.beta.)-/cycloartenol, 9,19-Cyclolanostan-3-ol,24-methylene-, (3.beta.)-/24-methylenecycloartanol and lanosterol, and the triterpene *β*-amyrin share a common precursor 2,3-oxidosqualene [35, 36] and together they make up a major part of the fraction which is 86.7%. In the Benz3 fraction cycloartenol and 24-methylenecycloartanol make up 70.7% of the fraction which is also a greater percentage that could have attributed to the observed single TLC band. In mammals, insects, and higher plants, sterols are also converted to steroidal hormones [37]; this could support the findings by Adegboye et al. [38] who found the presence of steroids in *Garcinia kola* seeds.

In all fractions cycloartenol was the most abundant compound followed by 24-methylenecycloartanol; the presence of these compounds is also supported by separate studies from other authors [17, 18] who found them present in *Garcinia kola* seeds' nonpolar solvent extracts. The commercially available compound of cycloartenol did not exhibit anti*Listeria* activities *in vitro* and this is also in line with the studies by Madubunyi [18], whereby the isolated cycloartenol did not exhibit antibacterial activities *in vitro*. In a study by Ragasa et al. [39] the cycloartenol isolated from the plant *Artocarpus heterophyllus* also showed no antibacterial activities but, however, exhibited antifungal activities.

The lack of anti*Listeria* activity from the most abundant compound in this study is not surprising as the beneficial medicinal effects of plant materials are sometimes not attributed to a single compound but to a combination of the compounds in it [3]. This could then suggest that the observed bioactivities were a result of the fractions' compounds acting in synergy. A higher test concentration than 5 mg/mL of the compound could have been needed to effect anti*Listeria* activity, but this concentration would have been too high for the compound to be classified as antimicrobial, since activity of pure plant compounds is routinely classified as being antimicrobial on the basis of susceptibility tests that produce lower MIC values in the range of 0.1 mg/mL to 1 mg/mL against the target organism [40].

Bioactivity portrayed by nonpolar extracts such as n-hexane is often associated with complex mixtures of triterpenoid and/or steroid compounds [41]. Anti*Listeria* activities exhibited by the ethanolic extracts of *Eremophila alternifolia* and *Eremophila duttonii*, in food homogenates and milk, were attributed to the presence of terpenes and/or sterols in the plants [42]. This could support the anti*Listeria* activities of the Benz2 fraction which had the sterols cycloartenol, 24-methylenecycloartanol and lanosterol, and **β**-amyrin which is a typical pentacyclic triterpene. Cycloartenol is a precursor of all plant sterols whilst lanosterol is the precursor of all mammals and yeasts sterols [37]. Some evidence indicates that the biosynthetic pathway of sterols via lanosterol also exists in plant cells [37], which could therefore explain the presence of lanosterol in the Benz2 fraction.

Synergistic and additive interactions are a result of a combined effect of the active compounds from the extracts and the antibiotics [27]. Both the Benz2 and Benz3 fractions had 25% synergistic and 75% additive interactions with ciprofloxacin, a fluoroquinolone antibiotic that inhibits DNA synthesis [43]. Penicillin G had 50% synergistic and 50% additive interactions with Benz3 fraction whilst it had 25% additive and 75% indifference interactions with the Benz2 fraction. However, ampicillin had 100% indifferent interactions with Benz2 whilst, with Benz3, it had 25% synergistic, 25% additive, and 50% indifferent interactions. It is possible that the presence of the other compounds in the Benz2 fraction which are absent in the Benz3 fraction such as lanosterol and *β*-amyrin caused more indifference interactions to be observed between the Benz2 fraction and the penicillins in comparison to the Benz3 interactions with the penicillins.

The mechanism of action of most lipophilic compounds against bacteria is postulated to be mainly due to their interference effects on the structural and functional properties of the bacterial membrane resulting in the membrane losing its integrity and becoming more permeable [44], such that the observed synergistic and additive interactions could be due to the lipophilic nature of the sterols in the fractions causing loss of membrane integrity, thereby, in the case of ciprofloxacin, allowing its increased and easier entry into the bacterial cell to cause DNA disruption, while in the case of the penicillins this resulted in the combination of the sterols' loss of membrane integrity properties and the penicillins' cell wall inhibiting properties producing a cumulative and/or enhanced bactericidal effect. The effects, however, were not consistent in all the test *Listeria* species as some of the isolates may have acquired resistance mechanisms due to different environmental exposures before they were isolated from the wastewater effluents.

The differences observed between how penicillin G and ampicillin interact with the same fraction could be due to the differences in their molecular structures. The penicillins consist of a thiazolidine ring connected to a *β*-lactam ring attached to a side chain. The side chain determines many of the pharmacologic characteristics of given penicillin. The presence of an amino group on the benzyl side chain of ampicillin distinguishes ampicillin from penicillin G [45].

## 5. Conclusion

The sterols were the major compounds found in both the Benz2 and Benz3 fractions that exhibited anti*Listeria* activities *in vitro*. The observed anti*Listeria* activities are highly likely to have been a result of the synergistic interactions of the compounds in the fractions. The interactions of the fractions and the chosen penicillins which are the first choice antibiotics of treatment for human listeriosis showed varying interactions from synergy to indifference. The fractions did not show any antagonistic interactions with the antibiotics which is a good indication of its potential in combination therapy although *in-vivo* tests are necessary to determine the feasibility of this hypothesis and also to determine the fractions' toxicity in relation to the doses that may be required. Its highly likely, however, that no deleterious side effects would be experienced from the use of these fractions in anti*Listeria* chemotherapy due to the long-term traditional medicinal use of the *Garcinia kola* seed in its origins of West and Central Africa.

## Figures and Tables

**Table 1:** Minimum inhibitory concentrations (MICs) of the eluted column chromatography fractions in mg/mL and the antibiotics in *μ*g/mL.

Antibacterial agent	*L. monocytogenes* (LAL 8)	*L. ivanovii* (LEL 30)	*L. ivanovii* (LEL 18)	*L. grayi* (LAL 15)
Benz1	Not active	Not active	Not active	Not active
Benz2	1.25	0.625	0.625	0.625
Benz3	2.5	0.625	1.25	1.25
Ciprofloxacin	0.313	0.313	0.313	0.625
Penicillin G	0.079	0.156	0.020	0.020
Ampicillin	0.079	0.039	0.079	0.079

**Table 2:** Identification of trimethylsilyl (TMS) derivatised compounds in the Benz2 fraction of *Garcinia kola* seeds.

Retention time	Number	Compound identity	Area %
23.815	1	9,19-Cyclolanost-24-en-3-ol, (3.*β*.)	39.943
24.394	2	9,19-Cyclolanostan-3-ol, 24-methylene-, (3.*β*.)	23.423
22.963	1	9,19-Cyclolanost-24-en-3-ol, (3.*β*.)	7.095
24.120	3	2(1H)-Phenanthrenone, 3,4,4a,4b,5,6,7,8,10,10a-decahydro-1,1,4a,7,7-pentamethyl-,[4aR-(4a.alpha.,4b.beta.,10a.*β*.)]	6.895
23.149	4	Lanosterol	6.758
23.445	5	.*β*.-Amyrin	2.949
23.367	1	9,19-Cyclolanost-24-en-3-ol, (3.*β*.)	2.653
25.080	6	N-Methyl-1-adamantaneacetamide	2.408
22.545	1	9,19-Cyclolanost-24-en-3-ol, (3.*β*.)	2.072
23.541	1	9,19-Cyclolanost-24-en-3-ol, (3.*β*.)	1.828
20.674	7	1-(2,4-Dihydroxybenzoyl)-3-ethyl-5-trifluoromethyl-5-hydroxy-2-pyrazoline	1.384
21.861	8	4-Methyl-2-trimethylsilyloxy-acetophenone	1.233
25.932	8	4-Methyl-2-trimethylsilyloxy-acetophenone	1.121
20.442	9	Z-8-Pentadecen-1-ol acetate	0.238

Total			99.99

**Table 3:** Identification of trimethylsilyl (TMS) derivatised compounds in Benz3 fraction in the n-hexane extract of *Garcinia kola* seeds.

Retention time	Number	Compound identity	Area %
23.659	1	9,19-Cyclolanost-24-en-3-ol, (3.*β*.)	46.041
24.245	2	9,19-Cyclolanostan-3-ol,24-methylene-, (3.*β*.)	24.695
24.017	3	Trimethyl[4-(1,1,3,3,-tetramethylbutyl)phenoxy]silane	7.711
23.093	4	Tetrasiloxane, decamethyl	7.396
22.909	5	5-Methyl-2-trimethylsilyloxy-acetophenone	6.904
23.398	6	2,4,6-Cycloheptatrien-1-one, 3,5-bis-trimethylsilyl	2.530
23.324	7	4-Methyl-2-trimethylsilyloxy-acetophenone	2.494
22.522	6	2,4,6-Cycloheptatrien-1-one, 3,5-bis-trimethylsilyl	2.230

Total			100

**Table 4:** Interactions of the Benz2 column chromatography fraction and some antibiotics.

Organism	Antimicrobial combination	FIC index of (antibiotic)	FIC index of Benz2	ΣFIC index	Interaction
*L. grayi* (LAL 15)	Benz2/Pen	1	0.063	1.06	Indifferent
	Benz2/Cipro	0.125	0.5	0.625	Additive
	Benz/Ampi	1	0.250	1.25	Indifferent

*L. monocytogenes* (LAL 8)	Benz2/Pen	1	0.250	1.25	Indifferent
	Benz2/Cipro	0.5	0.5	1	Additive
	Benz2/Ampi	1	0.250	1.25	Indifferent

*L. ivanovii* (LEL 18)	Benz2/Pen	1	0.063	1.06	Indifferent
	Benz2/Cipro	0.250	0.5	0.750	Additive
	Benz/Ampi	1	0.250	1.25	Indifferent

*L. ivanovii* (LEL 30)	Benz2/Pen	0.5	0.250	0.750	Additive
	Benz2/Cipro	0.125	0.250	0.370	Synergy
	Benz2/Ampi	1	0.250	1.25	Indifferent

Key: Pen denotes penicillin G, Ampi denotes ampicillin, and Cipro denotes ciprofloxacin.

**Table 5:** Interactions of the Benz3 column fraction of *Garcinia kola* seeds and some antibiotics.

Organism	Antimicrobial combination	FIC index of antibiotic	FIC index of Benz3	ΣFIC index	Interaction
*L. grayi* (LAL 15)	Benz3/Pen	0.5	0.031	0.531	Additive
	Benz3/Cipro	0.125	0.5	0.625	Additive
	Benz3/Ampi	1	0.250	1.24	Indifferent

*L. monocytogenes* (LAL 8)	Benz3/Pen	0.5	0.063	0.563	Additive
	Benz3/Cipro	0.25	0.25	0.5	Synergy
	Benz3/Ampi	0.497	0.063	0.560	Additive

*L. ivanovii* (LEL 18)	Benz3/Pen	0.25	0.016	0.266	Synergy
	Benz3/Cipro	0.125	0.5	0.625	Additive
	Benz3/Ampi	0.248	0.063	0.311	Synergy

*L. ivanovii* (LEL 30)	Benz3/Pen	0.25	0.25	0.5	Synergy
	Benz3/Cipro	0.125	0.501	0.626	Additive
	Benz3/Ampi	1	0.251	1.25	Indifferent

Key: Pen denotes penicillin G, Ampi denotes ampicillin, and Cipro denotes ciprofloxacin.
